# The Impact of Congenital Anomalies of the Male and Female Reproductive Organs on Infertility and Recurrent Pregnancy Loss: A Review

**DOI:** 10.3390/medicina62050812

**Published:** 2026-04-24

**Authors:** Bojana Petrovic, Sanja Kostic, Ivana Milan Jovanovic, Milica Petronijevic, Milos Petronijevic, Igor Hudic, Svetlana Vrzic Petronijevic

**Affiliations:** 1 Clinic for Gynecology and Obstetrics, University Clinical Center of Serbia, 11000 Belgrade, Serbia; 2 Faculty of Medicine, University of Belgrade, 11000 Belgrade, Serbia; 3 Clinic of Gynecology and Obstetrics, University Clinical Center, 75000 Tuzla, Bosnia and Herzegovina

**Keywords:** congenital reproductive tract anomalies, male and female reproductive anomalies, infertility, recurrent pregnancy loss

## Abstract

Congenital anomalies of the reproductive system represent a heterogeneous group of structural and functional abnormalities affecting both male and female genital organs. These anomalies typically arise during embryogenesis and may remain asymptomatic until they are incidentally identified during evaluation for infertility, recurrent pregnancy loss, or disorders of sexual development. In females, abnormalities include Müllerian duct anomalies and congenital malformations of the uterus, cervix, vagina, and ovaries, such as Mayer–Rokitansky–Küster–Hauser syndrome, septate, unicornuate, bicornuate, and didelphys uteri, and ovarian agenesis and undescended ovaries. In males, congenital conditions such as anorchia, cryptorchidism, hypospadias, ejaculatory duct obstruction, and ejaculatory dysfunction may be associated with impaired spermatogenesis and reduced fertility. Early recognition of these conditions may facilitate timely clinical evaluation and individualized management, which can include surgical correction, hormonal therapy, and reproductive counseling. When appropriate, early diagnosis may support multidisciplinary care, with the aim of optimizing sexual development, preserving reproductive potential, and reducing long-term morbidity associated with congenital anomalies. However, the clinical impact of early detection varies depending on the type and severity of the anomaly. A systematic and multidisciplinary approach may contribute to improved reproductive outcomes and better overall reproductive health in affected individuals. Further research is needed to better define the optimal timing and clinical utility of systematic evaluation strategies in this patient population.

## 1. Introduction

Sexual development represents a complex, multidimensional, and lifelong process through which biological sex characteristics and psychosexual attributes progressively emerge. It encompasses somatic and endocrine maturation as well as the development of gender identity and gender-role behavior, reflecting the interaction between genetic, hormonal, and psychosocial determinants [1]. Within this developmental continuum, disorders of sex development (DSD) are recognized as congenital conditions that arise from disruptions in sex differentiation and are therefore closely related to abnormalities of gonadal and reproductive tract development.

Within this framework, congenital anomalies of the reproductive system constitute an important group of conditions that may disrupt normal reproductive function and significantly influence both physical and psychosocial well-being. These anomalies are recognized as a major cause of adverse reproductive outcomes, with their clinical impact largely depending on the type and extent of anatomical distortion. While some affected individuals may retain normal fertility and sexual function, others experience substantial reproductive impairment, including amenorrhea, severe dysmenorrhea, preterm delivery, and, most notably, recurrent miscarriage and infertility [2].

Infertility, defined as the failure to achieve pregnancy after 12 months of regular unprotected sexual intercourse, affects a considerable proportion of couples, with an identifiable cause present in approximately 85% of cases. The most common etiologies include ovulatory dysfunction, male factor infertility, and tubal disease, whereas the remaining cases are classified as unexplained infertility [3]. Among the spectrum of reproductive disorders, recurrent pregnancy loss (RPL), defined as the loss of two or more pregnancies, represents a distinct and particularly challenging clinical entity, affecting approximately 1–2% of couples [4].

This narrative review aims to summarize and synthesize current evidence regarding the impact of congenital anomalies of both male and female reproductive organs on infertility and recurrent pregnancy loss (RPL), with a particular focus on their clinical implications.

## 2. Method

This study represents a narrative review of the current literature on congenital anomalies of the reproductive organs and their association with infertility and recurrent pregnancy loss (RPL). A structured search was conducted across multiple electronic databases, including PubMed, Medline, Cochrane Library, Google Scholar, Scopus, Web of Science, and EBSCO, covering studies published up to January 2026. The search strategy combined Medical Subject Headings (MeSH) and free-text terms, including “male reproductive organ,” “female reproductive organ,” “congenital anomaly,” “infertility,” and “recurrent pregnancy loss,” along with related keywords. Search terms were used both individually and in various combinations using Boolean operators to ensure comprehensive coverage of the literature.

The literature search was performed using predefined search terms and was conducted systematically across all databases to ensure comprehensive coverage of the available evidence. The final search was last updated in January 2026.

The scope of this review includes both male and female congenital anomalies affecting reproductive outcomes, encompassing anatomical, genetic, and developmental conditions.

The initial search yielded 192 records. After removal of duplicate entries, 157 articles remained and were screened by title and abstract. Two reviewers independently screened titles and abstracts, followed by full-text assessment. Discrepancies were resolved through discussion or consultation with a third reviewer. Studies available only as abstracts, those without accessible full texts, non-English publications, and articles not directly addressing the association between congenital anomalies and infertility or RPL were excluded.

Eligible studies included those addressing the epidemiology, pathophysiology, clinical presentation, reproductive outcomes, and management of congenital anomalies in both male and female reproductive systems. Priority was given to systematic reviews, prospective and observational studies, and contemporary clinical guidelines, while older landmark studies were included when necessary to provide important historical or mechanistic context. Reference lists of selected articles were also manually screened to identify additional relevant publications.

Due to significant heterogeneity in study design, populations, reported outcomes, and methodological quality, a quantitative meta-analysis was not feasible. Therefore, a narrative synthesis approach was adopted, with findings organized thematically to provide a comprehensive overview of the prevalence, clinical significance, and reproductive implications of congenital anomalies in both sexes. Although no formal risk-of-bias assessment tool was applied, study quality was appraised based on design, sample size, and consistency of findings, with greater emphasis placed on higher-level evidence. This review was conducted in accordance with the SANRA guidelines for narrative reviews.

## 3. Sexual Development in the Fetal Period

Sexual differentiation begins at fertilization with the establishment of chromosomal sex. The XX or XY chromosomal complement determines early gene expression patterns, producing molecular sex differences prior to the onset of morphological differentiation [5,6].

During the sixth week of embryogenesis, embryos remain sexually indifferent and possess two parallel ductal systems: the Wolffian (mesonephric) and Müllerian (paramesonephric) ducts [7]. Around the seventh post-fertilization week, differentiation of the bipotential gonadal ridge is initiated. Genetic signaling pathways direct its development into either testes or ovaries, thereby determining gonadal sex and establishing the endocrine framework for subsequent phenotypic differentiation [1].

In XY embryos, activation of the SRY gene on the Y chromosome triggers testicular organogenesis. This results in the differentiation of Sertoli cells, which organize seminiferous tubules, and Leydig cells, which synthesize testosterone. These cell populations collectively orchestrate male sexual differentiation. In XX embryos, the absence of SRY expression permits ovarian differentiation, characterized by folliculogenesis and the formation of oocytes essential for reproductive capacity [8].

From approximately the eighth week of gestation, differentiation of the internal reproductive tract and external genitalia proceeds in a hormone-dependent manner. In male embryos, testosterone and anti-Müllerian hormone (AMH) secreted by the testes promote persistence and transformation of the Wolffian ducts into the epididymis, vas deferens, and seminal vesicles, while AMH induces regression of the Müllerian ducts. In female embryos, the absence of these testicular hormones results in Müllerian duct persistence and differentiation into the fallopian tubes, uterus, and upper vagina, whereas the Wolffian ducts regress. The lower portion of the vagina develops from the urogenital sinus [9,10].

External genital development is likewise hormonally regulated. Androgen exposure drives masculinization of the genital tubercle and labioscrotal structures, leading to formation of the penis and scrotum. In the absence of significant androgenic stimulation, the clitoris, labia, and vaginal introitus develop [8].

Sex differentiation can therefore be conceptualized across three interdependent levels:

1. Chromosomal sex, established at fertilization (XX or XY);
2. Gonadal sex, determined by differentiation of the bipotential gonad during weeks 6–7 of embryogenesis;
3. Phenotypic Sex, Resulting from Hormonally Mediated Development of Internal and External Genital Structures [11].

### Disorders of Sexual Development

Disorders of Sexual Development (DSD) comprise a heterogeneous group of congenital conditions characterized by atypical development of internal and/or external genital structures, frequently associated with discordance among chromosomal, gonadal, and phenotypic sex. These conditions arise from genetic, hormonal, or developmental disruptions, including sex chromosome abnormalities, mutations in genes essential for embryonic sexual differentiation, and defects affecting hormone biosynthesis or action [12,13]. Clinical presentation varies considerably: some individuals are identified at birth due to ambiguous external genitalia, whereas others present later with postnatal virilization, delayed or absent puberty, or infertility.

The 2006 Consensus Statement categorizes DSD into three principal groups according to karyotype and underlying pathophysiology. 46,XY DSD includes disorders of testicular development, such as partial or complete gonadal dysgenesis and ovotesticular DSD, as well as abnormalities of androgen synthesis (e.g., 5α-reductase type 2 deficiency) or androgen action (partial or complete androgen insensitivity syndrome). 46,XX DSD encompasses disorders of ovarian development and conditions associated with androgen excess, most notably congenital adrenal hyperplasia (CAH). Sex chromosome DSD refers to variations in the typical sex chromosome complement, including Klinefelter syndrome and its variants (47,XXY), Turner syndrome and its variants (45,X), mosaic karyotypes (45,X/46,XY), and chimerism (46,XX/46,XY) [14,15].

Disrupted gonadal development constitutes a central pathogenic mechanism in most forms of DSD, underscoring the importance of understanding gonadogenesis when evaluating long-term outcomes, particularly reproductive potential [16]. Individuals with DSD may experience reduced fertility due to intrinsic subfertility associated with abnormal gonadal formation, progressive gonadal insufficiency, prophylactic gonadectomy, or anatomical constraints. Although these conditions were historically regarded as synonymous with infertility, emerging evidence indicates that some individuals retain biological fertility potential [17]. Recent research suggests that, under appropriate experimental protocols, a proportion of individuals previously presumed infertile may achieve fertility, particularly when fertility preservation strategies are implemented during childhood. Continued investigation is required to expand reproductive options for individuals with DSD, addressing not only clinical and scientific challenges but also patient perspectives, decision-making processes, and the optimization of surgical approaches [18].

## 4. Congenital Reproductive Tract Anomalies

Congenital anomalies of the male and female genital tract constitute a heterogeneous group of structural abnormalities affecting internal and/or external genitalia, arising during embryogenesis and present at birth. These conditions exhibit considerable variation in prevalence and severity, ranging from minor anatomical variations to complex malformations with substantial implications for reproductive and sexual health. They may affect different segments of the genital tract and represent deviations from typical anatomical configurations. Importantly, such anomalies can significantly impact reproductive outcomes, including fertility, pregnancy, and parturition [19].

Reproductive tract malformations generally result from aberrant development or arrest at critical stages of embryogenesis. Additionally, they may arise secondary to gene mutations, chromosomal abnormalities, hormonal imbalances, environmental exposures during gestation, or infections occurring during organogenesis [20].

A thorough understanding of congenital genital tract anomalies is essential in reproductive medicine, urology, gynecology, and obstetrics, given their diagnostic, therapeutic, and prognostic significance.

## 5. Congenital Anomalies of the Female Reproductive Tract

Congenital malformations of the female genital organs constitute a diverse group of anatomical variants that may result in functional impairments, often due to partial or complete obstruction of reproductive structures. These anomalies arise from errors in embryologic development, which can be driven by genetic mutations, hormonal disturbances, or environmental insults. Although many cases remain asymptomatic, they are clinically significant due to their potential impact on fertility and, in certain instances, female reproductive identity.

The prevalence of female reproductive tract anomalies in the general population is estimated at 3–7%, but this proportion rises markedly in specific clinical contexts, affecting up to 16% of women with recurrent miscarriage and up to 27% of women presenting with infertility or recurrent pregnancy loss [21,22,23,24,25]. Clinical manifestations and reproductive consequences vary according to the type and extent of anatomical distortion, which can range from complete vaginal agenesis to defects in the lateral or vertical fusion of the Müllerian ducts [26].

Disruptions in the development or fusion of the Müllerian ducts during embryogenesis may result in a broad spectrum of congenital abnormalities collectively referred to as Müllerian duct anomalies (MDAs). These anomalies involve all structures derived from the paramesonephric (Müllerian) ducts, including the fallopian tubes, uterus, and upper portion of the vagina [27]. MDAs are frequently identified during infertility evaluations and are clinically significant because of their impact on reproductive outcomes. Epidemiological studies indicate that Müllerian duct abnormalities are present in approximately 16% of women with recurrent pregnancy loss, highlighting their relevance in reproductive medicine [28,29].

### 5.1. Developmental Abnormalities of the Uterine Tubes

Tubal factor infertility (TFI) represents a major contributor to female infertility and requires thorough evaluation. The fallopian tubes, typically measuring 10–12 cm in length, consist of four segments extending from the uterus to the ovary: intramural, isthmic, ampullary, and fimbrial. Their structural integrity and functional competence are critical for oocyte capture, fertilization, and embryo transport [30].

Although relatively uncommon, congenital and anatomical abnormalities of the uterine tubes can markedly compromise reproductive potential. Reported anomalies include complete or partial (segmental) agenesis, accessory fallopian tubes, supernumerary ostia, and paratubal cystic formations, such as hydatids of Morgagni [27,31]. In rare instances, unilateral absence of a fallopian tube has been documented, occasionally in conjunction with ipsilateral ovarian agenesis [32].

These developmental abnormalities are often asymptomatic and frequently remain undetected until incidentally identified during imaging, laparoscopy, laparotomy, or cesarean delivery. Beyond congenital defects, TFI more broadly encompasses functional or structural impairment, including tubal obstruction or failure of the fimbrial apparatus to effectively capture the oocyte. Overall, TFI is one of the most prevalent causes of female infertility, accounting for up to 30–40% of cases [33].

Management of TFI depends on the site and extent of the obstruction. Proximal tubal lesions may be treated with tubal cannulation under hysteroscopic or laparoscopic guidance to restore patency. Isthmic obstructions are most commonly addressed via tuboplasty procedures. In cases of large hydrosalpinx with distal tubal damage, salpingectomy followed by in vitro fertilization (IVF) is generally recommended. Recanalization procedures for secondary infertility have a reported success rate of approximately 85% [33].

### 5.2. Congenital Uterine Anomalies

Congenital uterine anomalies (CUAs) represent structural defects of the uterus resulting from impaired embryological development of the Müllerian ducts, including disruptions in duct formation, fusion, resorption, or, in rare cases, duplication. These anomalies have been consistently associated with adverse reproductive outcomes, particularly infertility and pregnancy loss [34].

**Figure 1:**
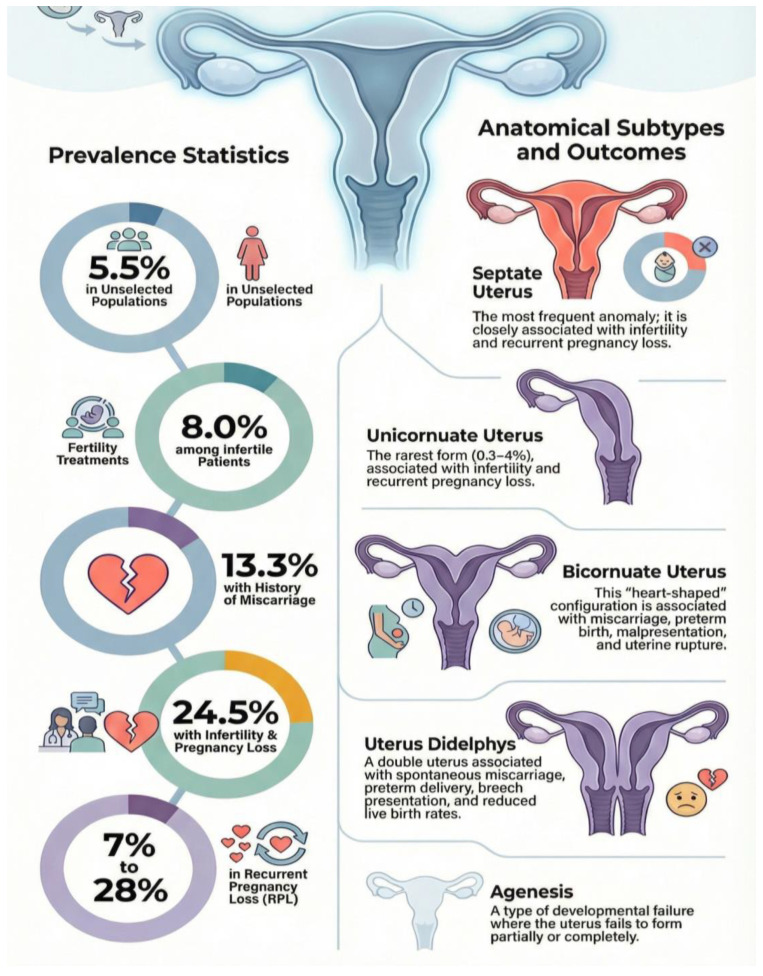
Congenital uterine anomalies (CUAs)—prevalence in different populations and their impact on fertility and pregnancy outcomes [35,36]. This figure is intended for illustrative and educational purposes. Due to heterogeneity in study populations, methodologies, and classification systems, the presented data should not be interpreted as directly comparable or as reflecting uniform levels of evidence across studies.

Epidemiological studies report considerable variability in prevalence, largely influenced by differences in study populations and diagnostic methods. In unselected populations, CUAs are identified in approximately 5.5% of women. Prevalence increases among women with reproductive disorders, reaching 8.0% in infertile patients, 13.3% in those with a history of miscarriage, and up to 24.5% in women presenting with both infertility and pregnancy loss [35] (Figure 1).

Analyses specifically addressing recurrent pregnancy loss (RPL) demonstrate heterogeneous findings. Reported prevalence of CUAs among women with RPL ranges from 7% to 28% [36] (Figure 1). A meta-analysis encompassing multiple studies found congenital uterine defects in 12.6% of patients with recurrent miscarriage [37]. These discrepancies likely reflect variations in classification systems, diagnostic accuracy, and inclusion criteria across studies.

Clinically, many congenital uterine anomalies remain asymptomatic and are frequently detected incidentally during routine gynecological evaluation. However, they are also commonly identified during investigations for infertility or recurrent pregnancy loss [38].

Among anatomical subtypes, the unicornuate uterus—resulting from unilateral Müllerian duct developmental failure—represents the rarest form, accounting for approximately 0.3–4% of all uterine anomalies. In contrast, bicornuate uteri have been reported in around 3.2% of fertile women [39,40]. Uterine configurations associated with increased risk of RPL include septate, unicornuate, bicornuate, didelphys, and arcuate uteri, with the septate uterus being the most commonly encountered congenital anomaly (Figure 1)

#### 5.2.1. Mayer–Rokitansky–Küster–Hauser Syndrome

Müllerian agenesis (MA), also referred to as Müllerian aplasia or complete uterine aplasia, is a rare congenital anomaly characterized by absence of the uterus in individuals with a normal female karyotype (46,XX), preserved secondary sexual characteristics, and normal external genitalia. Its reported incidence is approximately 1 per 4500–5000 females [41,42]. In the literature, the terms MA and Mayer–Rokitansky–Küster–Hauser (MRKH) syndrome are often used interchangeably, although subtle distinctions have been described.

MRKH syndrome is conventionally classified into two clinical subtypes. Type I (isolated form) is defined by uterine agenesis in the presence of normal ovaries and absence of associated extragenital malformations. Type II comprises uterine absence accompanied by additional anomalies involving the fallopian tubes, ovaries, and urinary tract. A more severe variant within this spectrum is the MURCS association (Müllerian duct aplasia, unilateral renal agenesis, and cervicothoracic somite anomalies) [43]. Epidemiological data indicate that Type I accounts for approximately 56–72% of cases, whereas Type II is observed in 28–44% of affected individuals [42].

Clinically, patients most commonly present during adolescence with primary amenorrhea. MRKH syndrome is identified in approximately 16% of individuals evaluated for primary amenorrhea, making it the second most frequent underlying cause after ovarian insufficiency [44].

The precise etiology remains incompletely understood, reflecting the complexity of genetic and embryological mechanisms governing Müllerian duct development. Familial clustering has been reported, with first-degree relatives exhibiting a 1–5% risk of congenital uterine anomalies, consistent with a multifactorial inheritance pattern. Most analyses support an autosomal dominant mode of transmission with sex-limited expression in females, suggesting potential paternal transmission of pathogenic variants [45].

Advances in assisted reproductive technologies (ART) have expanded options for affected individuals. Gestational surrogacy and uterine transplantation (UTx) have emerged as potential strategies to achieve biological parenthood. UTx involves grafting the uterus along with the cervix, supporting ligaments, and vascular structures to re-establish pelvic anatomical continuity. Reported successful pregnancies and live births following UTx indicate that this approach may enable reproductive outcomes comparable to natural gestation under appropriate clinical, ethical, and legal frameworks [46].

#### 5.2.2. Septate Uterus

Septate uterus is the most common congenital uterine malformation, accounting for approximately 35% of all diagnosed uterine anomalies [47,48]. It arises from defective embryological development, specifically incomplete resorption of the midline septum following fusion of the Müllerian ducts, resulting in partial or complete partitioning of the uterine cavity. Morphologically, septa exhibit considerable variability, with incomplete (partial) septa being most frequently observed.

Among uterine malformations, septate uterus has been consistently associated with unfavorable reproductive and obstetric outcomes. It is linked to infertility and is more commonly observed in infertile women compared with other uterine anomalies, although the precise pathophysiological mechanisms remain incompletely understood [45]. Quantitative analyses indicate reduced fertility in affected women (risk ratio [RR]: 0.86; 95% confidence interval [CI]: 0.77–0.96), alongside a significantly increased risk of miscarriage (RR: 2.9; 95% CI: 2.0–4.1) and preterm birth (RR: 2.1; 95% CI: 1.5–3.1) [49].

From an obstetric perspective, septate uterus is associated with particularly poor outcomes, with reported fetal survival rates ranging from 6% to 28% and spontaneous miscarriage rates exceeding 60% [50]. Pregnancy losses typically occur between 8 and 16 weeks of gestation and are often accompanied by clinical features suggestive of preterm labor.

Importantly, the uterine septum is considered the most common structural abnormality in women with recurrent pregnancy loss (RPL). Although historically described as the only potentially correctable congenital uterine defect in this context, the evidence supporting routine surgical correction remains limited and predominantly derives from retrospective studies [51]. Some observational studies suggest that hysteroscopic septum incision may reduce subsequent miscarriage rates and improve live birth rates in women with a history of RPL or infertility [52]. However, high-quality prospective randomized trials are lacking, and the benefit of septum resection may be influenced by patient selection and reproductive history. Evidence is particularly inconclusive in women with secondary infertility, emphasizing the need for careful individualized assessment and counseling based on current guidelines and observational data [53].

#### 5.2.3. Unicornuate Uterus

Unicornuate uterus (uterus unicornis) is a specific type of Müllerian duct anomaly resulting from unilateral developmental failure of one duct. It accounts for approximately 5–20% of all congenital uterine malformations. In some cases, a rudimentary horn is present, representing a rare anatomical variant with an estimated incidence of approximately 1 per 100,000 women.

Population-based data indicate a higher prevalence of unicornuate uterus among infertile women compared with the general population, with reported rates of approximately 0.5% in infertile cohorts versus 0.1% in the general population [33]. Overall, the anomaly affects an estimated 0.3% of women in the general population and represents 5–13% of all Müllerian anomalies [54].

Anatomically, the unicornuate uterus is classified into two main subtypes: (1) a form associated with a rudimentary horn containing a functional cavity—either communicating or non-communicating with the primary uterine cavity—and (2) a form without a rudimentary cavity. The condition is frequently asymptomatic and often detected incidentally during evaluation for infertility or adverse reproductive outcomes [54].

Reduced fertility observed in women with unicornuate uterus may be related to diminished uterine muscle mass and compromised vascularization, which can negatively affect implantation and pregnancy maintenance [55]. Moreover, affected women exhibit a significantly increased risk of early miscarriage, with a reported risk ratio of 2.15 (95% CI: 1.3–4.5) [49].

A substantial proportion of patients present with a functional, non-communicating rudimentary horn, which carries an elevated risk of rudimentary horn pregnancy and spontaneous abortion. Gestation within a non-communicating rudimentary horn is exceptionally rare but associated with severe complications, particularly uterine rupture. Standard management involves surgical removal of the rudimentary horn, often via laparoscopic resection in the first trimester of pregnancy [56].

Additional obstetric complications in this population include cervical insufficiency, preterm birth, fetal growth restriction, malpresentation, and abnormal placentation [57]. Despite generally less favorable reproductive outcomes, successful pregnancies are achievable. An overall successful pregnancy rate of 85.7% has been reported, with particularly favorable outcomes among women with secondary infertility (100%) [28]. These findings highlight the importance of early diagnosis and individualized obstetric surveillance to minimize risks and optimize management of potential complications.

#### 5.2.4. Bicornuate Uterus

A bicornuate uterus is a congenital uterine malformation subclassified into uterus bicornis bicollis and uterus bicornis unicollis, depending on whether the division occurs at the level of the external cervical os or above it. This anomaly accounts for approximately 10% of Müllerian duct anomalies [58].

Embryologically, a bicornuate uterus arises from partially incomplete lateral fusion of the paramesonephric (Müllerian) ducts around the tenth week of intrauterine development, primarily at the level of the uterine fundus. Consequently, two symmetrical uterine horns are formed, which are fused inferiorly and typically communicate at the level of the uterine isthmus [59]. Morphologically, this configuration results in two separate yet communicating endometrial cavities and usually a single cervix [60].

Epidemiological data suggest that bicornuate uterus occurs in fewer than 0.5% of females and constitutes approximately 10% of Müllerian duct anomalies. Uncomplicated pregnancies in women with this malformation are relatively uncommon [61]. The anomaly is associated with elevated risks of adverse obstetric outcomes, including miscarriage, preterm birth, malpresentation, and uterine rupture [60].

In non-pregnant women, bicornuate uterus has been linked to increased risks of infertility, endometriosis, painful hematometra, and urinary tract anomalies. During pregnancy, this malformation may predispose to spontaneous abortion, preterm delivery, cervical insufficiency, and is considered a potential risk factor for uterine rupture [62].

Most women with a bicornuate uterus remain asymptomatic, and the diagnosis is often established incidentally during pregnancy, in the context of obstetric complications, or at the time of hysterectomy. When identified prior to conception, surgical correction may be considered. Strassman metroplasty is an established reconstructive procedure aimed at unifying the uterine cavity. Conversely, when diagnosed during pregnancy, management is primarily expectant, guided by close clinical surveillance. In cases of cervical shortening, which is frequently observed in women with bicornuate uterus, cervical cerclage may be recommended to prolong gestation and reduce the risk of late miscarriage or preterm birth [63].

#### 5.2.5. Uterus Didelphys

Uterus didelphys, commonly referred to as a “double uterus,” is a rare congenital anomaly characterized by the presence of two completely separate uterine cavities, each with its own cervix. In up to 30% of cases, the condition is associated with a longitudinal vaginal septum [64].

Embryologically, uterus didelphys results from failure of fusion of the Müllerian ducts between the 12th and 16th weeks of fetal development. This disruption leads to the independent formation of two uterine horns, two cervices, and frequently duplication of the vagina. In most affected individuals, the anomaly remains clinically silent, often delaying diagnosis until reproductive age. However, some patients may present with symptoms such as dyspareunia and/or dysmenorrhea [65].

Uterus didelphys accounts for approximately 8% of congenital anomalies of the female reproductive tract. Its prevalence in the general population is estimated at around 0.3% [66,67]. Among infertile women, reported prevalence ranges from 0.2% to 0.3%, whereas in women with recurrent pregnancy loss, it varies between 0.6% and 2.1% [67]. In populations of women with a history of abortion and infertility, the frequency may reach 2.1% [66].

Reproductive outcomes in women with uterus didelphys are generally less favorable compared with those with normal uterine anatomy. Evidence indicates lower clinical pregnancy rates, reduced live birth rates, and an increased incidence of first-trimester pregnancy loss. This anomaly is also associated with elevated risks of obstetric complications, necessitating careful antenatal monitoring. Reported adverse outcomes include spontaneous miscarriage, preterm delivery, breech presentation, and overall reduction in live births relative to women with a structurally normal uterus [68]. Preterm birth rates have been estimated between 17.44% and 33.3% [65].

Despite these risks, successful pregnancies and deliveries have been documented, particularly following appropriate preconception surgical management when indicated and with targeted obstetric interventions such as cervical cerclage. Some women with uterine anomalies may also experience uncomplicated pregnancies or remain undiagnosed even after recurrent pregnancy losses.

Cesarean section is not routinely indicated solely on the basis of uterus didelphys and is generally reserved for standard obstetric indications [69].

### 5.3. Congenital Cervical Abnormalities

#### 5.3.1. Cervical Agenesis

Cervical agenesis is an extremely rare congenital anomaly, with a reported prevalence of approximately 1 in 80,000–100,000 women [70,71]. Vaginal agenesis coexists in about 39% of cases, whereas cervical agenesis with a normally developed vagina and functional uterus is even rarer, occurring in only 4.8% of affected individuals [71,72]. This malformation results from either abnormal fusion of the Müllerian ducts with the urogenital sinus or atrophy of a normally formed segment of the Müllerian system [72].

Delayed diagnosis may increase the risk of endometriosis and compromise normal tubal and ovarian function. Imaging modalities, including transvaginal ultrasonography and magnetic resonance imaging, allow precise characterization of the anomaly and facilitate effective preoperative planning [73].

Historically, treatment often involved hysterectomy or abdominal cervicovaginoplasty. With the advent of laparoscopy and advanced surgical techniques, minimally invasive procedures have become the preferred approach. Contemporary management focuses not only on symptom relief but also on preserving fertility, although reproductive potential remains limited due to structural compromise of the uterus and fallopian tubes [70,74].

#### 5.3.2. Congenital Cervical Atresia and Hypoplasia

Congenital cervical atresia and hypoplasia are rare anomalies resulting from abnormal development of the Müllerian system. These conditions may arise due to defective fusion of the Müllerian ducts with the urogenital sinus, incomplete canalization of the lower Müllerian tract, or segmental atrophy of a normally developed Müllerian structure. They can occur in isolation or in association with other genital tract malformations [75].

Management of these abnormalities typically requires reconstructive or extirpative procedures to relieve outflow tract obstruction, and infertility is a common consequence [76].

#### 5.3.3. Isolated Cervical Aplasia

Isolated cervical aplasia is an extremely rare congenital malformation, with an estimated incidence of approximately 1 in 100,000 births. This anomaly represents a disruption of the female genital outflow tract and is an inevitable cause of infertility. Affected patients commonly present with pelvic pain or haematometra, necessitating surgical intervention [73].

The pathogenesis of isolated cervical aplasia remains incompletely understood, although some authors propose that local atrophy at the level of the primitive cervix may contribute to the defect [77]. Cervical atresia has been further classified into several subtypes: (i) intact cervical body with obstruction of the cervical os, (ii) cervical body consisting of a fibrous band, (iii) fragmented cervical portions, and (iv) hypoplastic midportion of the cervix with a bulbous tip. Accurate assessment of the cervical remnant using imaging modalities is essential, as reconstructive procedures are guided by the observed anatomy [78].

The primary objectives of reconstructive surgery are to establish a functional conduit for menstruation, alleviate pain, and preserve reproductive potential. Despite surgical correction, the likelihood of achieving spontaneous pregnancy remains limited [78].

#### 5.3.4. Cervical Incompetence

Cervical incompetence (CI), also referred to as cervical insufficiency, is defined as the inability of the uterine cervix to maintain an intrauterine pregnancy in the absence of uterine contractions or overt labor. It typically presents as painless cervical dilatation due to structural or functional cervical abnormalities. CI is a recognized cause of recurrent pregnancy loss (RPL), particularly during the second trimester. The true incidence remains uncertain, as diagnosis is primarily clinical and lacks definitive objective criteria [79].

Cervical incompetence may be congenital or acquired. Among congenital causes, abnormalities in embryological development of the Müllerian ducts are considered the most frequent underlying factor [80].

Diagnosis is generally established based on obstetric history, most commonly recurrent second-trimester pregnancy loss preceded by spontaneous rupture of membranes and/or painless cervical dilatation. Currently, no validated objective diagnostic tests exist to reliably identify cervical weakness in non-pregnant women [81].

In pregnant women at increased risk, transvaginal ultrasonography is employed for surveillance. Sonographic findings suggestive of CI include a shortened cervical length (≤25 mm) and/or cervical funneling, defined as protrusion of the fetal membranes through a dilated internal cervical os while the external os remains closed [80].

Management of cervical insufficiency primarily involves two therapeutic approaches. Administration of vaginal progesterone reduces the incidence of preterm delivery in women with a shortened cervix, independent of prior preterm birth history. Cervical cerclage is similarly effective, particularly in patients presenting both a short cervix and a previous preterm birth [82].

### 5.4. Congenital Vaginal Abnormalities

#### 5.4.1. Congenital Vaginal Agenesis

Congenital vaginal agenesis is a rare developmental anomaly, with an estimated incidence of 1 in 4000–10,000 live female births [38]. In the majority of cases, it coexists with uterine agenesis and forms part of the clinical spectrum referred to as Müllerian aplasia, Müllerian agenesis, or Mayer–Rokitansky–Küster–Hauser (MRKH) syndrome. A functional uterus has been found in approximately 2–7% of patients with MRKH syndrome [83].

Embryologically, the vagina develops from two distinct structures: the upper two-thirds originate from the Müllerian ducts, whereas the lower third derives from the urogenital sinus. Disruption of Müllerian duct development can result in agenesis or atresia of the uterus, vagina, or both. The overall prevalence of congenital vaginal absence in the general population has been estimated at 0.001–0.025% [84,85].

Vaginal malformations are infrequently isolated and are often accompanied by additional congenital anomalies, particularly of the renal and skeletal systems. They may also occur within broader syndromic contexts, including Bardet–Biedl syndrome, Fraser syndrome, and MRKH syndrome.

Clinically, affected individuals typically present during adolescence with primary amenorrhea despite normal secondary sexual characteristics. Depending on the extent of associated anomalies, patients may also exhibit varying degrees of uterovaginal malformation, infertility, and concomitant renal, vertebral, or skeletal abnormalities [84].

#### 5.4.2. Vaginal Atresia

Vaginal atresia is an uncommon congenital anomaly of the female reproductive tract resulting from aberrant development of the urogenital sinus and Müllerian ducts, leading to incomplete canalization of the vaginal lumen. Its estimated incidence ranges from 1 per 10,000 to 15,000 live female births. In most cases, affected individuals present with normal external genitalia and preserved uterine, tubal, and ovarian morphology, while exhibiting partial or complete obstruction of the vaginal canal [86,87].

Isolated vaginal aplasia is considered to arise from defective development of the distal segments of the paramesonephric (Müllerian) ducts [88]. Clinically, vaginal atresia should be differentiated from other causes of outflow tract obstruction, particularly imperforate hymen and Mayer–Rokitansky–Küster–Hauser (MRKH) syndrome.

Initial diagnostic suspicion is typically based on clinical presentation and gynecological examination. The most common manifestations include primary amenorrhea, cyclic lower abdominal or pelvic pain, palpable pelvic masses due to hematocolpos or hematometra, and partial or complete absence of the vaginal introitus [89].

Vaginal atresia is frequently accompanied by additional congenital anomalies, which may involve other Müllerian duct malformations, such as unicornuate or septate uterus; urogenital defects including renal dysplasia and congenital vesicovaginal fistula, as well as extragenital anomalies such as scoliosis, congenital cardiac defects, polydactyly, and anal atresia [89].

Complete congenital vaginal atresia is often associated with cervical malformations, although uterine development is generally preserved and the endometrium remains functional. Symptom onset may therefore be delayed, with less pronounced abdominal pain and subtler pelvic masses, potentially postponing medical evaluation [90]. Progressive menstrual retention can lead to complications such as hematosalpinx, hemoperitoneum, and pelvic endometriosis. Notably, endometriosis has been reported in 66.7% of patients with distal vaginal hypoplasia and in all patients with combined cervical and vaginal underdevelopment [91].

Accordingly, even when the vagina is surgically reconstructed (i.e., a neovagina is created), infertility may still be a concern.

#### 5.4.3. Obstructed Hemi-Vagina with Ipsilateral Renal Agenesis (OHVIRA)

Obstructed hemivagina with ipsilateral renal agenesis (OHVIRA) syndrome, formerly referred to as Herlyn–Werner–Wunderlich syndrome, is a congenital malformation of the female urogenital tract [92]. Its reported incidence ranges from 0.1% to 3.8% [93]. OHVIRA is classified as an obstructive Müllerian anomaly and is defined by a triad consisting of uterine duplication (didelphys, bicornuate, or septate uterus), obstructed hemivagina, and ipsilateral renal agenesis or other renal anomalies [94].

Clinically, patients most commonly present with pelvic pain, vaginal bleeding, and compressive symptoms related to the underlying anatomical abnormalities, which may substantially impair quality of life and reproductive outcomes [95]. Due to the complexity of this syndrome, accurate diagnosis and timely management are important in order to reduce symptoms and improve reproductive prognosis. Fertility-preserving surgical intervention is generally considered the first-line approach in women of reproductive age, with reported favorable reproductive and obstetric outcomes following treatment [96].

These considerations highlight the clinical relevance of the association between Müllerian duct anomalies and renal abnormalities. In patients with suspected or confirmed congenital reproductive tract anomalies, evaluation of the urinary tract may be considered, and conversely, assessment of Müllerian structures may be warranted in patients with renal anomalies, as part of an individualized diagnostic approach.

### 5.5. Anomalies of Ovarian Development

Normal gonadal development depends on the proper migration of primordial germ cells and the adequate formation of the urogenital ridge. These processes are orchestrated by multiple genes and signaling pathways, and disruption at any stage of this tightly regulated sequence may result in unilateral or complete failure of ovarian development [97].

#### 5.5.1. Ovarian Agenesis

Ovarian agenesis (OA) is an exceptionally rare condition in obstetrics and gynecology, defined as the complete absence of one or both ovaries. Bilateral forms are exceedingly uncommon and are typically associated with profound endocrine and reproductive consequences, whereas unilateral absence is reported more frequently. Although precise incidence data are limited, available estimates suggest an occurrence of approximately 1 in 11,240–11,241 cases [98,99].

The etiology of OA is not fully elucidated but is generally explained by three principal mechanisms: embryological developmental defects, vascular compromise, or adnexal torsion followed by resorption of ovarian tissue [98]. Congenital cases may result from localized abnormalities of the genital ridge or caudal portion of the Müllerian duct [100]. Genetic factors, including chromosomal abnormalities such as Turner syndrome and X chromosome mutations, have also been implicated [99]. Additionally, early angiogenesis is essential for physiological ovarian development, and impairment in this process may contribute to agenesis [100].

Unilateral ovarian absence (UOA) represents the more common presentation of OA. It is characterized by the absence of one ovary, often accompanied by partial or complete absence of the ipsilateral fallopian tube and/or adnexa, and occasionally associated with additional genital or extragenital anomalies [98]. Unlike bilateral forms, unilateral cases may remain asymptomatic and are frequently identified incidentally during imaging or infertility evaluation.

Clinical manifestations vary depending on whether one or both ovaries are absent. Bilateral ovarian agenesis typically presents with primary amenorrhea, delayed pubertal development, and deficiencies in estrogen, progesterone, and testosterone. Some affected individuals may exhibit tall stature and low body weight [101]. In contrast, unilateral agenesis may preserve partial ovarian function, resulting in milder or absent endocrine disturbances.

Diagnostic assessment relies primarily on imaging modalities, including ultrasound (US) and magnetic resonance imaging (MRI), which are critical for confirming ovarian absence and identifying associated anomalies. In patients presenting with amenorrhea and hormonal insufficiency, hormone replacement therapy has proven effective in restoring menstruation, improving bone mineral density, and supporting weight gain [99].

#### 5.5.2. Undescended Ovaries

Undescended ovary (UO) is an uncommon gynecological anomaly characterized by the localization of the superior adnexal pole above the level of the common iliac vessels. The condition is rarely reported, primarily through isolated case studies. Due to the shared embryological development of the genital and urinary systems, UO is frequently associated with Müllerian and renal tract malformations. Clinical diagnosis based solely on presentation is challenging, as symptoms are generally nonspecific, and approximately 60% of patients remain asymptomatic [102].

The estimated incidence of UO is approximately 0.3%. It is generally classified into two forms: cases occurring in conjunction with congenital uterine anomalies and isolated forms without associated uterine malformations. Maldescent of the ovary and fallopian tube is more commonly observed in patients with uterine abnormalities; Müllerian agenesis has been reported in up to 20% of these cases, while a unicornuate uterus is identified in approximately 40% [103].

Many patients are diagnosed incidentally during infertility evaluation. When clinical manifestations are present, they are often vague, leading to misdiagnosis and delayed recognition, with definitive identification frequently occurring during surgical exploration [102].

Although ectopic ovaries are most commonly detected in the context of infertility assessments, their direct impact on reproductive capacity remains uncertain [104]. Anatomical maldescent alters the spatial relationship between the ovary and fallopian tube, potentially hindering oocyte capture by the fimbriae, fertilization, or embryo transport toward the uterine cavity, thereby possibly compromising reproductive outcomes [105].

Overall, congenital reproductive tract anomalies represent a heterogeneous group of conditions with variable clinical significance in the context of infertility and recurrent pregnancy loss. The strength of evidence supporting their association with adverse reproductive outcomes differs substantially across specific anomalies.

Congenital uterine anomalies, particularly septate uterus, are supported by the most robust evidence base, including observational studies and meta-analyses demonstrating consistent associations with infertility and recurrent pregnancy loss. Other Müllerian anomalies, such as unicornuate, bicornuate, and uterus didelphys, are also associated with increased risks of adverse obstetric outcomes; however, the available evidence is predominantly derived from observational studies and is therefore best interpreted as moderate in certainty.

In contrast, evidence regarding cervical, vaginal, and ovarian developmental anomalies remains more limited and is largely based on retrospective series, case reports, and small cohort studies. Accordingly, the extent to which these conditions independently contribute to infertility and recurrent pregnancy loss is less well established and may be considered low in certainty. For rare entities such as ovarian agenesis and undescended ovary, the current literature is primarily descriptive, and conclusions regarding reproductive impact remain largely inferential.

Taken together, congenital reproductive tract anomalies should be regarded as clinically relevant findings in the evaluation of reproductive dysfunction; however, the heterogeneity in study design and evidence quality necessitates cautious interpretation of their relative contribution to infertility and recurrent pregnancy loss across different conditions.

## 6. Congenital Anomalies of the Male Reproductive Tract

Male fertility is defined as the ability to achieve conception through the production of viable and functionally competent spermatozoa, effective deposition of sperm within the female reproductive tract, and successful fertilization of the oocyte. The causes of male infertility may be congenital or acquired and are commonly classified based on the level of dysfunction as pre-testicular, testicular, or post-testicular [106].

Men with congenital genitourinary (GU) anomalies—including cryptorchidism, hypospadias, and other structural abnormalities of the external genitalia—are at increased risk of impaired spermatogenesis. In certain cases, underlying genetic defects contribute both to the anatomical malformation and to subsequent reproductive dysfunction, potentially resulting in infertility or recurrent pregnancy loss (RPL) [107].

The reported prevalence of congenital external genital abnormalities in males ranges from 3.4% to 18.7%. Among these conditions, phimosis is the most frequently observed, followed by inguinal hernia, hypospadias, hydrocele, and undescended testes [108].

### 6.1. Anorchia

Congenital anorchia—also referred to as vanishing testis syndrome or functional prepubertal castration—is a rare condition characterized by the complete absence of one or both testes in individuals with a normal male phenotype and a 46,XY karyotype. The estimated prevalence is approximately 1:20,000 for bilateral cases and 1:5000 for unilateral involvement. The etiology remains incompletely defined and is thought to be multifactorial [109].

Approximately 50% of neonates with bilateral anorchia present with micropenis, suggesting prenatal disruption of testicular development or maintenance. Clinically, affected individuals exhibit primary hypogonadism before puberty, including delayed pubertal progression, eunuchoid body proportions, markedly reduced serum testosterone concentrations within the castrate range, and compensatory elevations of gonadotropins. On physical examination, the testes are non-palpable, although remnants such as blind-ending spermatic cords or epididymides may occasionally be identified [110,111].

Histological evaluation may reveal microscopic residual structures—including seminiferous tubules or isolated germ cells—in a minority of patients (0–16%), with variable anatomical distribution [112]. Proposed pathophysiological mechanisms include intrauterine testicular torsion, vascular compromise, or trauma affecting the developing testes. Histopathological findings, such as hemosiderin-laden macrophages and dystrophic calcifications, support the concept of antenatal testicular regression. Genetic contributions are increasingly recognized, particularly in familial cases, suggesting that anorchia may lie within the broader spectrum of 46,XY disorders of gonadal development [113,114].

Diagnosis is supported biochemically by undetectable serum anti-Müllerian hormone (AMH) and inhibin B, alongside elevated follicle-stimulating hormone (FSH), in the context of a confirmed 46,XY karyotype. Initiation of low-dose testosterone therapy at the appropriate age facilitates normal development of secondary sexual characteristics and supports attainment of expected adult height [114].

Given the theoretical risk of malignant transformation in residual testicular remnants, some authors recommend laparoscopic exploration and excision of any identifiable tissue to mitigate long-term oncological risk [112].

### 6.2. Persistent Müllerian Duct Syndrome

Persistent Müllerian Duct Syndrome (PMDS) is a rare autosomal recessive disorder characterized by the presence of Müllerian duct derivatives—such as the uterus and fallopian tubes—in otherwise phenotypically male individuals. External genitalia are typically normal, and androgen-dependent virilization as well as testicular endocrine function are generally preserved.

PMDS is often diagnosed incidentally during surgical procedures for inguinal hernia or cryptorchidism. In a substantial proportion of cases, both testes are located on the same side, a presentation referred to as transverse testicular ectopia, and may be found adjacent to structures analogous to the broad ligament. Associated anomalies of the male excretory duct system are frequently observed. Although infertility is common, reproductive potential may be retained if at least one testis is descended into the scrotum and maintains intact ductal connections.

The majority of cases result from pathogenic variants in either the anti-Müllerian hormone (*AMH*) gene or the anti-Müllerian hormone receptor type 2 (*AMHR2*) gene [115]. Surgical management often focuses on orchidopexy to preserve testicular function, while careful consideration is given to the excision of Müllerian remnants to minimize the risk of malignancy without compromising fertility.

### 6.3. Cryptorchidism (Undescended Testis)

Cryptorchidism refers to the absence of one or both testes from the scrotal sac due to incomplete descent during fetal development and represents the most common congenital abnormality of the male genitourinary tract. Under normal circumstances, testicular descent into the scrotum is completed between the 25th and 35th weeks of gestation [116].

Cryptorchidism may present as either a unilateral or bilateral condition, with a higher frequency of involvement of the right testis. Bilateral cryptorchidism is observed in approximately 10% of patients with undescended testes [117]. The condition occurs in approximately 3% of full-term male neonates and in up to one-third of preterm infants.

Among individuals with undescended testes, the prevalence of monorchidism (unilateral absence of a testis) may reach up to 4%, whereas the prevalence of anorchidism (bilateral absence of the testes) is less than 1% [118]. The principal long-term consequences of cryptorchidism include subfertility or infertility and an increased lifetime risk of testicular germ cell tumors. Malignant transformation is attributed to prolonged suprascrotal exposure of the testicular tissue, while impaired fertility results from a multifactorial interplay of hormonal dysregulation, intrinsic testicular abnormalities, environmental exposures, and genetic susceptibility [119].

A strong association exists between a history of undescended testes and impaired spermatogenesis in adulthood. Approximately 10% of men presenting with infertility report a history of cryptorchidism treated by orchiopexy. Prior testicular maldescent has been documented in 20–27% of men with azoospermia and in 3–8% with oligo-terato-asthenospermia. In unilateral cases, the probability of infertility is estimated up to 32%, whereas bilateral involvement nearly doubles this risk even after surgical correction. In untreated bilateral cases, azoospermia occurs in almost 89% of individuals [120].

The etiology of cryptorchidism is multifactorial and incompletely understood. Familial aggregation observed in cryptorchidism and hypospadias suggests heritable contributions, although it remains unclear whether this reflects single-gene mutations, polygenic inheritance, epigenetic modifications, or shared environmental factors. Epidemiological studies also implicate paternal exposure to environmental toxins, including heavy metals and tobacco smoke, as potential risk factors for testicular maldescent in offspring [121].

At a molecular level, pathogenic variants in genes such as *INSL3* and *RXFP2* have been identified in select cases, highlighting the role of disrupted hormonal signaling in testicular descent. However, these mutations are rare and insufficient to explain the majority of cases, supporting the multifactorial or polygenic nature of the condition [121].

Bosma arhinia microphthalmia syndrome (BAMS) is an exceptionally rare congenital syndrome (~100 cases reported worldwide) that combines craniofacial malformations—such as complete absence of the external nose (arhinia) and ocular anomalies including microphthalmia, cataracts, and colobomas—with reproductive dysfunction. Affected males frequently exhibit hypogonadotropic hypogonadism, micropenis, cryptorchidism, incomplete pubertal development, and infertility. Heterozygous pathogenic variants in the *SMCHD1* gene have been implicated [122].

Early recognition and timely surgical intervention are critical to minimizing long-term reproductive and oncologic risks. Referral to a pediatric surgical specialist is recommended if spontaneous descent has not occurred by six months of age, if cryptorchidism is first diagnosed later, or if acute testicular torsion is suspected. Routine imaging, such as ultrasonography, is generally not recommended for initial evaluation, as it rarely alters management, while diagnostic laparoscopy is frequently required to confirm testicular location. Current guidelines advocate orchiopexy between 6 and 18 months of age.

Despite technically successful correction, fertility outcomes remain suboptimal in men with prior bilateral involvement. Individuals with a history of undescended testes also carry a higher incidence of testicular cancer compared with the general population. Both the original anatomical location of the testis and the timing of surgical intervention are important prognostic determinants for reproductive potential and malignancy risk. Postpubertal regular testicular self-examination is therefore advised to facilitate early detection of neoplastic changes [123].

### 6.4. Physical Factors

Physical factors may impair spermatogenesis or obstruct sperm transport through the ejaculatory ducts. In some cases, semen is redirected into the urinary bladder during ejaculation, a phenomenon termed retrograde ejaculation. This condition accounts for approximately 2% of male infertility cases and is frequently linked to structural or functional abnormalities of the bladder neck sphincter, particularly in patients with congenital urinary tract anomalies, including those involving the bladder [124,125].

### 6.5. Obstructive Azoospermia and Male Infertility

Obstructive azoospermia is a major cause of male infertility, affecting approximately 40% of men with azoospermia. It results from congenital or acquired obstructions within the male reproductive tract, including the vas deferens, epididymis, and ejaculatory ducts, with epididymal obstruction being the most common etiology. The prevalence of epididymal obstructive azoospermia (EOA) ranges from 42.4% to 48% [126].

#### 6.5.1. Epididymal Anomalies

Epididymal anomalies frequently arise from abnormal involution of the mesonephric (Wolffian) duct adjacent to the testis. Embryologically, the epididymal body and vas deferens originate from the mesonephric tubules and Wolffian duct, with the connection between the rete testis and mesonephric tubules established around 12 weeks of gestation [127]. Disjunction anomalies may result from differential mesonephric duct development or vascular alterations during tubule fusion [128] and are classified as disjunction anomalies, epididymal atresia, or elongated epididymis [129].

Although anomalies of the testis, epididymis, and vas deferens occur in roughly 2% of normal male fetuses, their prevalence is markedly higher in cryptorchid testes [130]. Additional defects involving the epididymis, vaginal process, and gubernaculum can further impair fertility by altering the anatomical relationships between the epididymis and testis. Among boys with cryptorchidism, these anomalies are reported in 16–75% of cases, reflecting variability in classification and detection [131].

Microsurgical vasoepididymostomy (MVE) remains the gold-standard treatment for EOA in patients seeking natural conception. While MVE can restore fertility, intraoperative challenges, including difficulties in separating or reconstructing the genital tract, may necessitate the use of assisted reproductive technologies (ART) to achieve pregnancy [126].

#### 6.5.2. Congenital Absence of the Vas Deferens

Congenital absence of the vas deferens (CAVD) occurs in 1–2% of infertile men and may present as either bilateral (CBAVD) or unilateral (CUAVD). CBAVD is frequently associated with congenital absence of the epididymal corpus and cauda, as well as dysplasia or agenesis of the seminal vesicles. It is often identified during evaluation for azoospermia and may represent a manifestation of cystic fibrosis or occur independently [132].

CUAVD arises from Wolffian duct developmental defects, with an incidence of 0.06–1% in the general male population and 0.08–1.25% in vasectomized men [133,134]. Unilateral renal agenesis (URA) is a common associated anomaly, reported in 19–85% of cases, reflecting the shared embryological origin of the renal collecting system and seminal excretory tract. Overall, CBAVD accounts for 1–2% of male infertility, while CUAVD contributes to 0.4–1% of cases [135].

Genetically, CBAVD is most often linked to mutations in the CFTR gene, although truncating mutations in ADGRG2 have also been reported [134,136]. Spermatogenesis is typically preserved; however, obstruction prevents sperm transport, necessitating assisted reproductive technologies, such as surgical sperm retrieval and in vitro fertilization, for biological parenthood [137,138]. Diagnosis is facilitated by transrectal and scrotal ultrasonography, particularly when physical palpation is inconclusive [139].

#### 6.5.3. Ejaculatory Duct Obstruction

Ejaculatory duct obstruction (EDO) is a rare cause of male infertility, affecting 1–5% of men evaluated for reproductive concerns [140,141]. It can result in aspermia, azoospermia, oligoasthenospermia, painful ejaculation, hematospermia, or infertility. Like the epididymis, vas deferens, and seminal vesicles, the ejaculatory ducts develop from the Wolffian duct [142]. Congenital causes include agenesis or atresia of the ejaculatory ducts, CFTR gene mutations, and ectopic ureteral openings, with fewer than 200 cases reported [143].

Clinical presentation depends on obstruction severity. Complete bilateral EDO produces low-volume (<1.5 mL), acidic, azoospermic ejaculate with absent or low fructose levels. Partial obstruction may present as oligo-, astheno-, or teratozoospermia, often with normal ejaculate volume and fructose content [143,144]. Some patients remain asymptomatic, while others experience perineal pain, painful ejaculation, or hematospermia.

Treatment traditionally involves transurethral resection of the ejaculatory ducts, which is effective but carries risk of surgical trauma and postoperative complications. Minimally invasive alternatives, including transrectal seminal vesicle imaging with balloon dilation and seminal vesiculoscopy, have been developed to reduce complications while preserving therapeutic efficacy [145].

**Figure 2:**
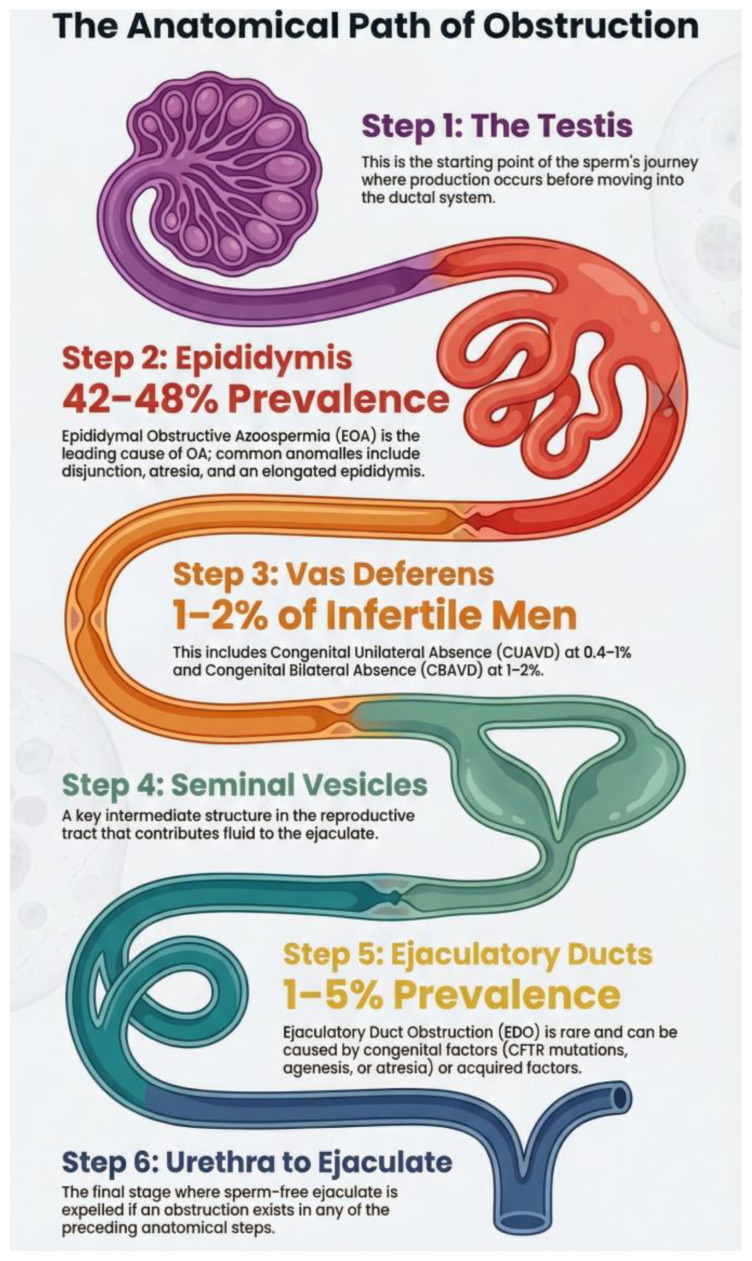
Obstructive sites in the male reproductive tract. Key points: epididymis is the most common obstruction site, often due to embryological anomalies; vas deferens obstruction can be congenital (CAVD) or acquired, with CUAVD frequently associated with ipsilateral renal agenesis; ejaculatory ducts are a rare but clinically significant site, obstruction can be congenital or acquired and may cause azoospermia or low ejaculate volume. Abbreviations: EOA—epididymal obstructive azoospermia; CAVD—congenital absence of the vas deferens; CBAVD—congenital bilateral absence of the vas deferens (CBAVD); CUAVD—congenital unilateral absence of the vas deferens; EDO—ejaculatory duct obstruction [126,135,140,141].

Congenital obstructions within the male reproductive tract are presented in Figure 2.

### 6.6. Hypospadias

Hypospadias is among the most common congenital anomalies of the male external genitalia and represents a significant aspect of pediatric urogenital pathology. Its global prevalence ranges from 0.2 to 6.4 per 1000 live male births, with some reports indicating an increasing incidence in recent decades [146]. As the second most frequent genitourinary malformation after cryptorchidism, hypospadias carries not only surgical relevance but also implications for long-term reproductive and endocrine health.

Embryologically, hypospadias results from disrupted morphogenesis of the urethral plate and incomplete fusion of the urethral folds along the ventral penile midline. Development of the male external genitalia begins around the eighth week of gestation with differentiation of the genital tubercle. Between weeks 11 and 16, androgen-dependent fusion of the urethral folds accompanies elongation, culminating in formation of the penile urethra. Interference with this hormonally regulated process leads to ectopic positioning of the urethral meatus and varying degrees of ventral penile curvature [107].

Clinically, hypospadias spans a spectrum of severity based on the anatomical location of the urethral opening. Distal forms, with the meatus on the glans, generally correspond to milder structural alterations. Midshaft and proximal variants involve more extensive ventral displacement toward the penoscrotal region or perineum and are often associated with pronounced chordee and abnormal preputial configuration. Greater proximal severity correlates with increased anatomical and functional impairment [147,148].

The etiopathogenesis is multifactorial, involving interactions between genetic predisposition and environmental factors. Endocrine-disrupting chemicals capable of anti-androgenic or estrogen-like effects during critical fetal periods have been implicated. Substances such as dioxins, furans, polychlorinated biphenyls, organochlorine pesticides, phthalates, brominated flame retardants, and certain heavy metals may interfere with androgen signaling essential for urethral closure [149].

Hypospadias is frequently accompanied by additional genitourinary anomalies. Up to 40% of affected individuals exhibit concomitant urinary tract abnormalities. The presence of hypospadias alongside cryptorchidism or micropenis warrants comprehensive evaluation for disorders of sex development, reflecting potential underlying endocrine or genetic disturbances.

Long-term reproductive outcomes are influenced by anatomical severity. Distal hypospadias generally exerts minimal effect on adult fertility, whereas proximal forms are associated with reduced semen volume, sperm concentration, total sperm count, motility, and abnormal morphology [150]. These findings highlight the importance of considering hypospadias not merely as a pediatric surgical concern but as a condition with potential reproductive implications into adulthood.

Fertility in patients with Hypospadias is generally preserved in mild forms, while more severe cases may be associated with impaired reproductive outcomes, primarily due to anatomical and associated testicular abnormalities rather than the urethral defect itself [151].

Management aims to restore urinary function, support normal sexual function, and optimize cosmetic results. Surgical repair is typically performed between six and eighteen months of age, though the optimal timing remains debated due to considerations of anesthetic exposure, tissue characteristics, complication rates, and psychosocial development. Despite generally favorable functional and esthetic outcomes, approximately one-quarter of patients require secondary procedures. Optimal care is best delivered through a multidisciplinary approach involving pediatric urology, neonatology, reconstructive surgery, endocrinology, genetics, and psychological support [150].

### 6.7. Congenital Genitourinary Anomalies as Determinants of Male Infertility and Recurrent Pregnancy Loss

Congenital abnormalities of the male genitourinary (GU) tract represent an important group of developmental conditions that may contribute to impaired male reproductive function and adverse reproductive outcomes, including recurrent pregnancy loss (RPL). Unlike acquired disorders, these anomalies often reflect underlying genetic and embryological disturbances that may affect testicular development, spermatogenesis, and sperm quality.

Men with congenital GU anomalies, including cryptorchidism, hypospadias, and other malformations of the external genitalia, may present with an increased risk of impaired semen parameters. These conditions are frequently associated with disrupted testicular development and may occur as part of syndromic or nonsyndromic phenotypes, suggesting that reproductive dysfunction can be one component of broader developmental abnormalities [107].

Cryptorchidism illustrates the potential long-term reproductive consequences of abnormal testicular development. Infertility in affected individuals is multifactorial and largely related to impaired spermatogenesis. Semen abnormalities have been reported in a proportion of men with unilateral and bilateral cryptorchidism, and persistent subfertility may occur even after surgical correction, indicating that early developmental disturbances likely play an important role in long-term reproductive outcomes [107].

Hypospadias, particularly in its more severe forms, has also been associated with altered semen quality in adulthood. Affected men may exhibit reduced semen volume and impaired sperm parameters, suggesting that abnormal genital development may coexist with or reflect underlying testicular dysfunction [107].

Beyond conventional semen parameters, some studies suggest that men from couples experiencing RPL may exhibit higher levels of sperm DNA fragmentation and altered chromatin integrity compared with controls, indicating a potential role of paternal genomic quality in early embryonic development and pregnancy maintenance [152,153]. However, these associations are multifactorial, and lifestyle and environmental factors may also contribute to sperm DNA damage [154,155].

Overall, current evidence suggests that congenital genitourinary anomalies may be associated with impaired male fertility and may contribute to adverse reproductive outcomes, including RPL, although the strength of this association and its causal contribution vary across conditions and studies. These anomalies may act in conjunction with genetic, environmental, and lifestyle factors to affect spermatogenesis and sperm genomic integrity. Therefore, evaluation of men in infertile or RPL couples may benefit from careful consideration of congenital reproductive tract anomalies and associated developmental or syndromic conditions.

Overall, congenital anomalies of the male reproductive tract represent a heterogeneous group of conditions with variable implications for male fertility and, to a lesser extent, recurrent pregnancy loss (RPL). The strength of evidence supporting their impact on reproductive outcomes differs substantially depending on the specific anomaly and underlying mechanism.

Among these conditions, cryptorchidism is supported by the most consistent body of evidence, including longitudinal cohort studies and meta-analyses demonstrating associations with impaired spermatogenesis, subfertility, and an increased risk of testicular malignancy. Similarly, congenital obstructive anomalies—such as congenital absence of the vas deferens (CAVD), epididymal anomalies, and ejaculatory duct obstruction—are well-documented causes of obstructive azoospermia and male infertility, supported by clinical, genetic, and surgical data, and therefore represent well-established contributors to impaired reproductive function.

In contrast, conditions such as hypospadias and persistent Müllerian duct syndrome (PMDS) are associated with reproductive dysfunction; however, the available evidence is predominantly derived from observational studies and heterogeneous patient populations. Accordingly, their impact on fertility is best interpreted as moderate in certainty and often depends on severity, associated anomalies, and underlying endocrine or genetic factors.

Rare entities, including congenital anorchia and other developmental disorders of testicular formation, are supported primarily by case series and descriptive studies. While their association with infertility is biologically plausible, the overall evidence base remains limited and the reproductive implications are not fully characterized.

Furthermore, emerging data suggest that congenital genitourinary anomalies may be associated with altered sperm DNA integrity and could contribute to adverse reproductive outcomes such as RPL. However, these findings are based on limited and heterogeneous studies, and causality remains difficult to establish due to the influence of confounding genetic, environmental, and lifestyle factors.

Taken together, congenital anomalies of the male reproductive tract should be regarded as clinically relevant in the evaluation of male infertility and possibly RPL. Nevertheless, significant variability in study design, population characteristics, and outcome measures necessitates cautious interpretation of their relative contribution across different conditions, with clear distinctions between better-established and more speculative associations.

## 7. Limitations

Several limitations should be acknowledged. First, this review is narrative in nature and does not follow a fully systematic methodology, and no standardized risk-of-bias assessment was performed. Second, a substantial proportion of the available evidence is derived from review articles, as well as retrospective, single-center studies, case reports, or case series, with considerable heterogeneity in study design, definitions, and reported outcomes. Third, only studies published in English were included, which may have introduced language bias. Furthermore, the variability across studies precluded quantitative synthesis, necessitating a descriptive approach.

In addition, the broad and heterogeneous scope of the included conditions, encompassing both congenital anomalies and disorders of sex development, may limit comparability and conceptual consistency. The available evidence is largely associative and does not allow firm conclusions regarding causality, particularly in the context of male factor contributions to reproductive outcomes. Moreover, the strength of evidence varies across conditions, with some associations supported primarily by limited data. Therefore, the clinical implications of these findings should be interpreted with caution and should not be considered as definitive recommendations.

These limitations may affect both the internal validity and the generalizability of the conclusions.

## 8. Conclusions

Congenital anomalies of the reproductive system in both sexes represent a significant, yet often underrecognized, cause of infertility and recurrent pregnancy loss. It should be emphasized that the presence of a congenital reproductive anomaly does not necessarily imply a causal relationship with infertility or recurrent pregnancy loss, but may represent a potential contributing factor, in accordance with the interpretative nature of this narrative review.

Although existing neonatal ultrasound screening frameworks, such as those used for developmental dysplasia of the hip, may provide a conceptual basis for potential future integration, the extension of such protocols to include routine assessment of the reproductive system remains investigational [156]. Neonatal or early-infant ultrasound screening for reproductive anomalies should therefore be considered a hypothesis-generating concept. Further prospective studies are needed to evaluate its potential clinical utility, including diagnostic performance, risk of overdiagnosis, psychological impact, and health-economic implications.

Early identification of congenital reproductive anomalies may potentially facilitate timely clinical management, including surgical intervention, hormonal therapy, and multidisciplinary supportive care when appropriate, with the aim of optimizing patient outcomes and reproductive health [157]. Similarly, early recognition of male reproductive tract anomalies may assist in identifying conditions such as anorchia or PMDS, allowing for earlier clinical evaluation and management.

A comprehensive, multidisciplinary approach involving gynecologists, urologists, endocrinologists, genetic counselors, and mental health professionals may support normal sexual development, appropriate maturation of secondary sexual characteristics, preservation of sexual function, and potentially more favorable reproductive outcomes.

## Data Availability

No new data were created or analyzed in this study. Data sharing is not applicable to this article.

## Abbreviations

The following abbreviations are used in this manuscript:

ADGRG2	Adhesion G Protein-Coupled Receptor G2
AMH	anti-Müllerian hormone
AMHR2	anti-Müllerian hormone receptor type 2
ART	Assisted reproductive technologies
BAMS	Bosma arhinia microphthalmia syndrome
CAH	Congenital adrenal hyperplasia
CAVD	Congenital absence of the vas deferens
CBAVD	Congenital bilateral absence of the vas deferens
CFTR	Cystic fibrosis transmembrane conductance regulator
CI	Cervical incompetence
CUA	Congenital uterine anomaly
CUAVD	Congenital unilateral absence of the vas deferens
DNA	Deoxyribonucleic acid
DSD	Disorders of Sexual Development
EDO	Ejaculatory duct obstruction
EOA	Epididymal obstructive azoospermia
FSH	Follicle-stimulating hormone
GU	genitourinary
INSL3	Insulin-like factor 3
IVF	In vitro fertilization
MA	Müllerian agenesis
MDA	Müllerian duct anomaly
MRI	magnetic resonance imaging
MRKH	Mayer–Rokitansky–Küster–Hauser syndrome
MURCS	Müllerian duct aplasia, unilateral renal agenesis, and cervicothoracic somite anomalies
MVE	Microsurgical vasoepididymostomy
OA	Ovarian agenesis
OHVIRA	Obstructed hemivagina with ipsilateral renal agenesis syndrome
PMDS	Persistent Müllerian Duct Syndrome
RPL	Recurrent pregnancy loss
RXFP2	Relaxin Family Peptide Receptor 2
SMCHD1	Structural Maintenance of Chromosomes Flexible Hinge Domain-containing protein 1
SRY	Sex-determining Region Y
TFI	Tubal factor infertility
UO	Undescended ovary
UOA	Unilateral ovarian absence
URA	Unilateral renal agenesis
US	ultrasonography
UTx	Uterine transplantation
